# A Novel Extended Granger Causal Model Approach Demonstrates Brain Hemispheric Differences during Face Recognition Learning

**DOI:** 10.1371/journal.pcbi.1000570

**Published:** 2009-11-20

**Authors:** Tian Ge, Keith M. Kendrick, Jianfeng Feng

**Affiliations:** 1 Centre for Computational Systems Biology, Fudan University, Shanghai, People's Republic of China; 2 Cognitive and Systems Neuroscience Group, The Babraham Institute, Cambridge, United Kingdom; 3 Centre for Scientific Computing, University of Warwick, Coventry, United Kingdom; University College London, United Kingdom

## Abstract

Two main approaches in exploring causal relationships in biological systems using time-series data are the application of Dynamic Causal model (DCM) and Granger Causal model (GCM). These have been extensively applied to brain imaging data and are also readily applicable to a wide range of temporal changes involving genes, proteins or metabolic pathways. However, these two approaches have always been considered to be radically different from each other and therefore used independently. Here we present a novel approach which is an extension of Granger Causal model and also shares the features of the bilinear approximation of Dynamic Causal model. We have first tested the efficacy of the extended GCM by applying it extensively in toy models in both time and frequency domains and then applied it to local field potential recording data collected from *in vivo* multi-electrode array experiments. We demonstrate face discrimination learning-induced changes in inter- and intra-hemispheric connectivity and in the hemispheric predominance of theta and gamma frequency oscillations in sheep inferotemporal cortex. The results provide the first evidence for connectivity changes between and within left and right inferotemporal cortexes as a result of face recognition learning.

## Author Summary

The right temporal cortex has previously been shown to play a greater role in the discrimination of faces in both sheep and humans. In the frequency domain, analysis of the relative causal contributions of low (theta 4–8Hz) and high (gamma 30–70Hz) frequency oscillations reveals that prior to learning, theta activity is more predominant in right than in left hemisphere processing, and that learning reduces this so that high frequency oscillations gain more control. We have been able to demonstrate that the frequency of connections increases in the right hemisphere and decreases between the left and right hemispheres after learning. The results are obtained based upon a way to combine aspects of both the Granger and Dynamic Causal Models, which can be used to establish significant causal relations in both time and frequency domains and applied to local field potential recordings from multiple (64 channel) electrodes implanted in the inferotemporal cortex of both sides of the brain in sheep in order to establish changes in causal connections within and between the two hemispheres as a result of learning to discriminate visually between pairs of faces. It is anticipated that this new approach to the measurement of causality will not only help reveal how the two brain hemispheres interact, but will also be applicable to many different types of biological data where variations in both frequency and temporal domains can be measured.

## Introduction

In order to exploit the full potential of high-throughput data in biology we have to be able to convert them into the most appropriate framework for contributing to knowledge about how the biological system generating them is functioning. This process is best conceptualized as first building a nodal network derived from empirically derived knowledge of the biological structures and molecules involved (nodes) and then secondly to use computational-based steps to discover the nature, dynamics and directionality of connections (directed edges) between them.

Causality analysis based upon experimental data has become one of the most powerful and valuable tools in discovering connections between different elements in complex biological systems [1]–[6]. However, despite some encouraging successes in various areas in systems or computational biology its development and application have been impeded by a number of issues about the meaning of causality. For example, in clinical science, the current emphasis on how to apply causality approaches mainly resides in resolving the problem of how clearly to define causality itself [7]. A typical problem cited is the so called “Simpson paradox” in which the successes of groups seem reversed when the groups are combined. This demonstrates the ambiguity that can result in determining causal relationships based only on frequency data. However, this issue disappears if we incorporate time into the definition of causality as Granger has done in the field of Economics [8]. Nevertheless there is still no accepted unified way to tackle this issue. Taking altered gene expression data using microarray analysis as an instance, there are three approaches one can use to deal with the time-series data obtained: the simple dynamical system approach, the dynamical Bayesian network approach and the Granger causality approach which is a generalization of the dynamical system one. In [9], we have discussed in detail the pros and cons of applying the latter two approaches and shown potential advantages in using Granger when sufficient repeated measurements are available. With brain activity data from functional magnetic resonance imaging (fMRI) experiments, two prominent techniques have been introduced to address temporal dependencies and directed causal influences: Dynamic Causal (DCM) and Granger Causal (GCM) models. These two models have always been considered to differ radically from each other [10],[11]. DCM establishes state variables in the observed data and is believed to be a causal model in a true sense. On the other hand, GCM is a phenomenological model which just tests statistical dependencies among the observations to determine how the data may be caused [10]–[12]. The importance of the two approaches in interpreting fMRI data is demonstrated in [10] and in 2008 there were around 450 papers published devoted to both approaches and excluding those relating to other types of biological data.

The key question we want to address in the current paper is whether we can develop an extended and biophysical constraint approach to share the features of the various approaches mentioned above, and in particular of the two causal models: DCM and GCM? The significance of such an approach is obvious and we would expect that its application could represent a powerful new tool in systems and computational biology, particularly in association with increasingly powerful genomic, proteomic and metabolic methodologies allowing time-series measurements of large numbers of putatively interacting molecules.

In this paper, we will show that GCM can be extended to a more biophysical constraint model by incorporating some features of the bilinear approximation of DCM. By setting up a conventional VAR model with additional deterministic inputs and observation variables, we can create a more general model: Extended Granger Causal Model (EGCM) which offers a new way to establish connectivity.

The EGCM is first tested in two toy models. With both state and observation variables, the interactions between nodes are successfully recovered using an extended Kalman filter approach and partial Granger to establish causality in both time (DCM) and frequency (GCM) domains respectively. The GCM approach itself is not tailored particularly well for biological experiments where we are often faced with the case of the data being recorded with and without a stimulus present. The time gap between two adjacent stimuli is very short and we would expect the network structure to remain unchanged during the whole experiment although the form and the intensity of the input may be unknown. This scenario is also the case for the gene network data considered in [1],[2], where the authors have treated the two situations separately, although there should be a common and true structure for both. We have therefore also used EGCM in toy models to establish its efficacy in revealing the true network structure when there is an intermittent input to affect state variables.

To exemplify the direct application of EGCM to establishing causality in a specific biological system, we have applied it to local field potential (LFP) data recorded in the sheep inferotemporal cortex (IT) of both left and right hemispheres before and after they learn a visual face discrimination task [13]. There is electrophysiological, molecular neuroanatomical and behavioral evidence for asymmetrical processing of faces in the sheep brain similar to humans [14]–[16] although cells in both the left and right IT respond selectively to faces [15]. Learning also alters both local and population based encoding in sheep IT as well as theta-nested gamma frequency oscillations in both hemispheres and there is greater synchronization of theta across electrodes in the right IT than there is in the left IT [13]. There is considerable interest in establishing functional differences between the ways the left and right brain hemispheres interact and process information [17]–[19]. It has recently been hypothesized that the left hemisphere specializes in controlling routine and tends to focus on local aspects of the stimulus while the right hemisphere specializes in responding to unexpected stimuli and tends to deal with the global environment [18],[19]. Establishing altered causal connections and frequency dependency within and between the two hemispheres during face recognition learning will help test this hypothesis.

## Methods

### Ethics Statement

All animal experiments were performed in strict accordance with the UK 1986 Animals Scientific Procedures Act (including approval by the Babraham Institute Animal Welfare and Ethics Committee) and during them the animals were housed inside in individual pens and able to see and communicate with each other. Food and water were available ad libitum. Post-surgery all animals received both post-operative analgesia treatment to minimize discomfort and antibiotic treatment to prevent any possibility of infection.

### EGCM Model

The traditional and widely used Granger Causal Model takes the form [20]:
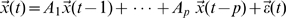
(1)where 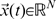, 
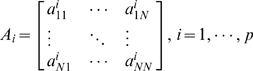
 are coefficient matrices, 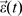 is the noise, and the model has a vector autoregressive representation with an order up to 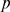.

In spite of its successful application, GCM requires the direct observation of the state variables and does not include designed experimental effects in the model which form some of its limitations. Here we extend GCM to a more reasonable and biophysical constraint model by incorporating additional deterministic inputs and observation variables, closely following equations which are the features in the Dynamical Causal Model and its bilinear approximation form [10]. The extended Granger Causal Model takes the form:
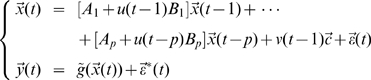
(2)where 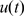 and 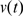 are deterministic inputs, 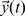 are the observation variables which are the function 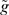 of the state variables, 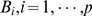 are the coefficients that allow the inputs to modulate the coupling of the state variables, 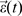 and 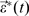 are intrinsic and observation noise and are mutually independent.

Now, if we can recover the state variables 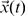 from the noise observation variables 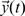, all the problems can be considered in the framework of the traditional Granger causality. It's clear that the formal difference between EGCM and GCM is that we have included observation variables and deterministic inputs which are assumed to be known and will affect the connection of the state variables as well as the state variables directly. However, EGCM also has a strong connection with Dynamical Causal Model. We refer the readers to Discussion section for a detailed discussion on the importance of this extension, in particular the relationship between Eq. (2) and the Volterra type series expansion [10].

### EGCM Algorithm

For the extended Granger Causal model Eq. (2), we now introduce an algorithm to estimate the state variables as well as all its parameters which will give us the first inspiration of the connection of the state variables.

Let 
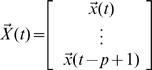
, 
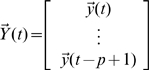
, 
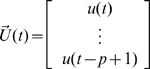
.

Then, the VAR(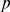) model can be reduced to a VAR(1) model which takes the form:
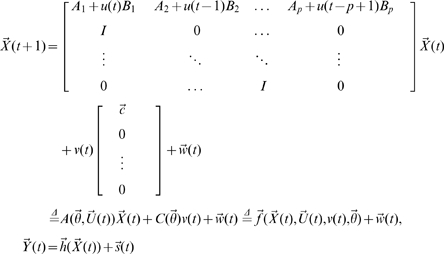
where 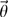 is the parameter vector to be estimated. 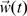 and 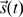 are both zero-mean uncorrelated Gaussian noise with covariance matrix 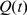 and 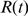 respectively.

In order to apply the model to real data, we have to estimate both the states and parameters of the model from input variables and noise observations. A widely used method for this dual estimation is extended Kalman filter (EKF) [21],. Here we recursively approximate the nonlinear system by a linear model and use the traditional Kalman filter for the linearized model.

Let 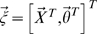 then
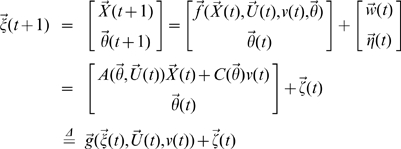
where 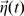 is uncorrelated Gaussian noise with covariance matrix 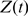. Define 
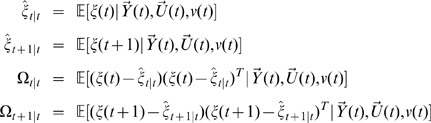
where 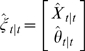, 
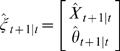
. Then, the EKF algorithm for dual estimation consists of two steps: prediction and updating.

#### Prediction

Given the estimated state 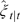, the observation 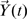 and inputs 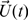 and 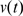, we predict the state variables and the covariance matrix of prediction error of the system at time 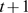.
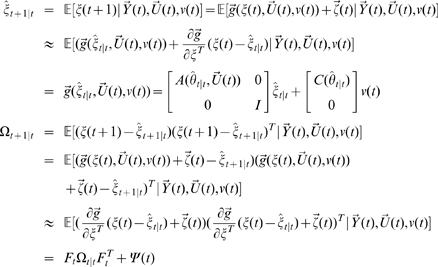
where 
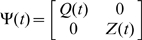
 and
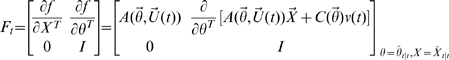



#### Updating

We use the new observation 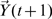 at time 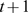 to update the state of the system.
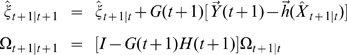
where
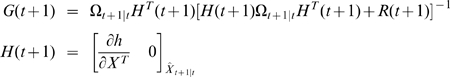



### EGCM Definition of Causality

After recovering the state variables using the EGCM algorithm above, we can define the causality with the idea proposed by Granger. The only difference is that, in our EGCM model, two deterministic inputs 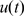 and 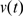 are added to the normal autoregressive representation. Here, we provide the formulation of EGCM causality in both time domain and frequency domains.

### Causality in the Time Domain

For simplicity of notation, here we only formulate EGCM for two time series 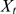 and 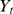. To generalize them to more general case of multi time series, we refer the readers to [23],[24]. Assume that 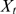 and 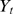 in our EGCM have the following representation:
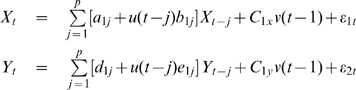
(3)A joint representation in our EGCM that includes the past information of both processes 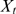 and 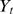 can be written as:
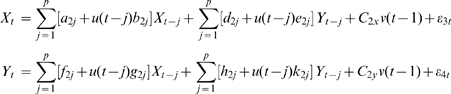
(4)where 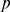 is the maximum number of lagged observations included in the model. 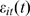, 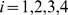 are prediction errors with variance 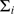 and are uncorrelated over time. Then, according to the causality definition of Granger, if the prediction of one process can be improved by incorporating the past information of the second process, then the second process causes the first process. So, in the extended model here, we define that if the variance of prediction error for the process 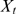 is reduced by the inclusion of the past information of the process 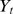, then, a causal relation from 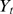 to 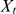 exists. This can be quantified as
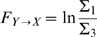
(5)If 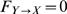, there is no causal influence from 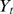 to 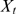 and if 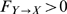, there is. Similarly, we can define the causal influence from 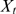 to 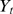 as
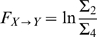
(6)


### Causality in the Frequency Domain

Our EGCM also allows a frequency domain decomposition to detect the intrinsic causal influence which provides valuable information.

We define the lag operator 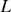 to be 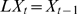 and assume here that the input 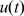 is a constant, i.e. 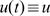 to avoid the appearance of nonlinearity. Then, the joint representation of both processes 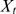 and 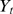 in equation (4) can be expressed as:
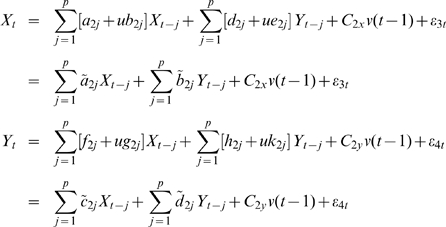
(7)


Rewrite equation (7) in terms of lag operator, we have:
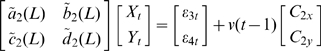
(8)where 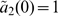, 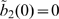, 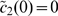, 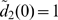.

Since what we really care about is the causal relationship caused by the intrinsic connection of the state variables rather than the outside driving force, i.e. the input 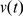, after fitting the model (7) and getting the covariance matrix of the prediction error, we just go on with the following model:
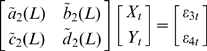
(9)which means that after fitting the EGCM with input 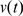 to eliminate outside influence, we just focus on the intrinsic causal influence in the frequency domain.

After normalizing equation (9) using the transformation proposed by Geweke [25],[26] to eliminate the cross term in the spectra, we assume that we have the normalized equation in the form:
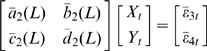
(10)Fourier transforming both sides of equation (10) leads to
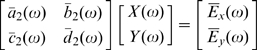
(11)Recasting equation (11) into the transfer function format we obtain
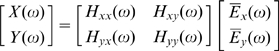
(12)After proper ensemble averaging we have the spectral matrix
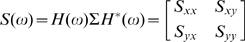
(13)where * denotes the complex conjugate and matrix transpose and 
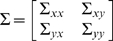
 is the covariance matrix of the prediction errors in equation (11). Hence, we can define the causal influence from 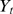 to 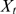 at frequency 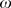 as
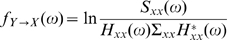
(14)Similarly, we can define the causal influence from 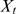 to 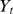 at frequency 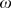 as well.

Note that although here we just provide the definition of pairwise Granger causality for EGCM, it's obvious that similar methods can be easily applied to the definition of conditional, partial or complex Granger causality in both time and frequency domains [9], [23], [24], [27]–[30]. Since the explicit meaning of the parameters in the EGCM (i.e. the intrinsic coupling among state variables, the strength of the inputs to modulate the coupling and the influence of the inputs on the state variables directly), we can also get an idea of the connection of the state variables and how the inputs affect them from the fitted model before we translate it into a single number.

### Methods for LFP Recording Experiment

#### Animals and visual discrimination training

Three female sheep were used (Ovis aries, one Clun Forest and two Dorsets). All experiments were performed in strict accordance with the UK 1986 Animals Scientific Procedures Act. During the experiments the animals were housed inside in individual pens. They were trained initially over several months to perform operant-based face (sheep) or non-face (objects) discrimination tasks with the animals making a choice between two simultaneously presented pictures, one of which was associated with a food reward. During stimulus presentations, animals stood in a holding trolley and indicated their choice of picture by pressing one of two touch panels located in the front of the trolley with their nose. The food reward was delivered automatically to a hopper between the two panels. The life-sized pictures were back projected onto a screen 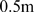 in front of the animal using a computer data projector. A white fixation spot on a black background was presented constantly in between trials to maintain attention and experimenters waited until the animals viewed this spot before triggering presentation of the image pairs. The stimulus images remained in view until the animal made an operant response (generally around 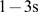). In each case, successful learning of a face or object pair required that a performance criterion of 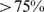 correct choices over 40 trials (i.e. 40 pairs) was achieved consistently. By the end of training, animals were normally able to reach the 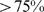 correct criterion after 40–80 trials and maintain this performance. For the current analysis extensive recordings taken during and after learning of novel face pairs were used (2 pairs for Sheep A; 2 pairs for Sheep B – only one of which was successfully learned – and 1 pair for Sheep C). In all cases recordings were made over 20–80 trials during learning and then during 46–170 trials after the 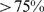 correct criterion was reached. Post learning trials ranged from within 5–10 minutes of the end of a learning trial session to 2 months after learning. For the face pairs, Sheep A and B were discriminating between the faces of different unfamiliar sheep faces (face identity discrimination) whereas for Sheep C, discrimination was between calm and stressed face expressions in the same animal (face emotion discrimination). With this latter animal, the calm face was the rewarded stimulus.

#### Electrophysiological recordings and analysis

Following initial behavioral training sheep were implanted under general anesthesia (fluothane) and full aseptic conditions with unilateral (Sheep A-right IT) or bilateral (Sheep B and C) planar 64-electrode arrays (epoxylite coated, etched, tungsten wires with 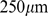 spacing - total array area around 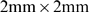 tip diameter 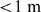, electrode impedence 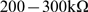) aimed at the IT. Holes (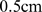 diameter) were trephined in the skull and the dura beneath cut and reflected. The electrode bundles were introduced to a depth of 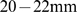 from the brain surface using a stereotaxic micromanipulator and fixed in place with dental acrylic and stainless-steel screws attached to the skull. Two of these screws acted as reference electrodes, one for each array. Electrode depths and placements were calculated with reference to X-rays, as previously described. Electrodes were connected to 34 pin female plugs (2 per array) which were cemented in place on top of the skull (using dental acrylic). Starting 3 weeks later, the electrodes were connected via male plugs and ribbon cables to a 128 channel electrophysiological recording system (Cerebus 128 Data Acquisition System - Cyberkinetics Neurotechnology Systems, USA) and recordings made during performance of the different face and non-face pair operant discrimination tasks. This system allowed simultaneous recordings of both neuronal spike and local event-related (LFP) activity from each electrode. Typically, individual recording sessions lasted around 30 min and for 80–200 individual trials. There was at least a week between individual recording sessions in each animal. The LFPs were sampled at 2kHz and digitized for storage from around 3 seconds prior to the stimulus onset to around 3 seconds after the stimulus onset (stimulus durations were generally 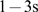).

For data analysis of the stored signals LFP data contaminated with noise such as from animal chewing food were excluded as were LFPs with unexpectedly high power. For LFPs, offline filtering was applied in the range of 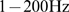 and trend was removed before spectral analysis. Any trial having more than 5 points outside the mean 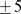 standard deviation range were discarded before analysis. At the end of the experiments, animals were euthanized with an intravenous injection of sodium pentobarbitone and the brains removed for subsequent histological confirmation of X-rays that array placements were within the IT cortex region.

## Results

In order to evaluate the performance of EGCM for the estimation of the state variables as well as the prediction of the parameters, we first applied the method to two toy models.

### Toy Models

#### Toy Model 1

The first toy model we used comes from a traditional VAR model which has been extensively applied in tests of Granger causality [31]. We modified the model by adding two deterministic inputs 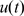 and 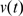. 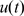 was assumed to be a constant stimulation, i.e. 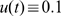 while 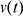 was assumed to a harmonic oscillator and had the form of a sinusoidal function since biological rhythms are a common phenomenon. Observation variables were also included in the toy model and assumed to be nonlinear functions of the state variables since it's a real challenge to uncover state variables with nonlinear mapping from states to measurements [32],[33]. We generated the time series according to the following equations:
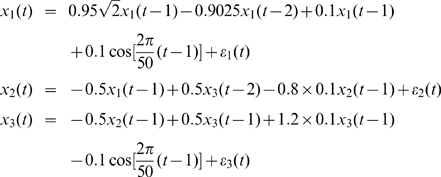
where 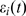, 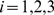 were zero mean uncorrelated Gaussian noise with variance 0.5, 0.8 and 0.6 respectively. Hence, according to the general form of EGCM, in this toy model we have:
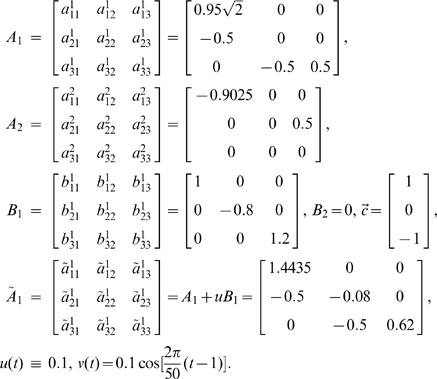
Inspection of the above equations reveals that 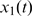 is a direct source to 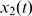, 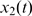 and 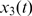 share a feedback loop. There is no direct connection between the remaining pairs of the state variables.

**Figure 1:**
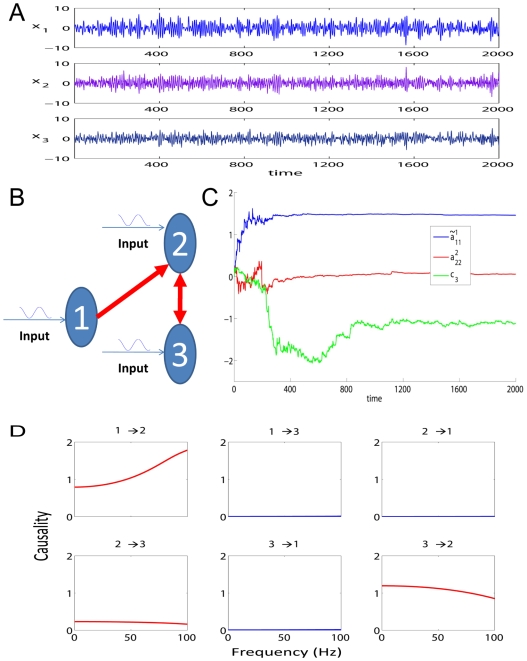
Results on Toy Model 1. A. Traces of the time series. B. The causal relationships considered in Toy Model 1 between the three state variables. C. The estimated parameters 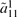, 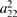, and 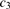 for the simulated data in Toy Model 1. The initial values of the three parameters are all set to 0. The covariance matrix 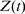 is first set to decay slowly to achieve faster convergence and then set to decay faster after two hundred time points to ensure a better accuracy. D. Frequency decomposition of all kinds of relationships between the state variables. Significant causal influences are marked by red.

Fig. 1A is an example of the 2000 time-steps of the data and Fig. 1B shows the network structure.

The observation variables were
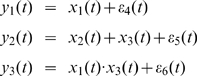
where 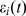, 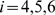 were zero mean uncorrelated Gaussian noise with variance 0.1 and also uncorrelated with 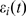, 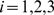.

Now, we can apply the method to this toy model, i.e., to estimate all the parameters 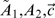 and state variables 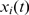 from the deterministic input 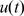, 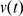 and noise observations. Simulations were performed for 2 seconds (2000 equally spaced time points). Fig. 1C shows that the parameters converged to their true values with only small fluctuations after several hundred data points, even though no prior knowledge was included and the initial values of the parameters were assigned to zeros. It has already been pointed out that the covariance matrix 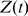 (see Methods section) of the noise in the parameter equation will affect the convergence rate and tracking performance [34]. In the situation here, a steep decay of the covariance matrix will lead to a better accuracy but the convergence is then slow. On the other hand, a slow decay will lead to a faster convergence but a larger fluctuation is observed. Hence, 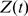 was carefully controlled to reduce to zero as the 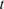 increased (see Fig. 1C).

After the state variables being recovered, we computed the partial Granger causality in both time and frequency domains (see Fig. 1D) and used the bootstrap approach to construct confidence intervals. Specifically, we simulated the fitted model to generate a data set of 1000 realizations of 2000 time points and use 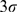 as the confidence interval. In this result, a causal connection was illustrated as part of the network if, and only if, the lower bound of the 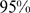 confidence interval of the causality was greater than zero. The results show that our extended model can detect the causal relationship correctly in both time and frequency domains.

#### Toy Model 2

When dealing with real data it is quite common that we need to detect the causal influence between time series from several variables affected by some stimulus. The stimulus may be very complicated, or hard to measure, and it may be impossible to formulate its form explicitly. However, if we ignore the influence of these inputs and use a traditional VAR model to detect the causality it is quite probable that we will get a misleading structure even if we use a high-order VAR model.

We used the following toy model which has exactly the same connection coefficients between the three state variables considered in Toy model 1 with an additional simple constant input function 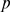:
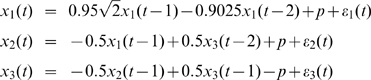
(15)where 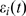, 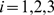 were zero mean uncorrelated Gaussian noise with variance 0.5, 0.8 and 0.6 respectively.

**Figure 2:**
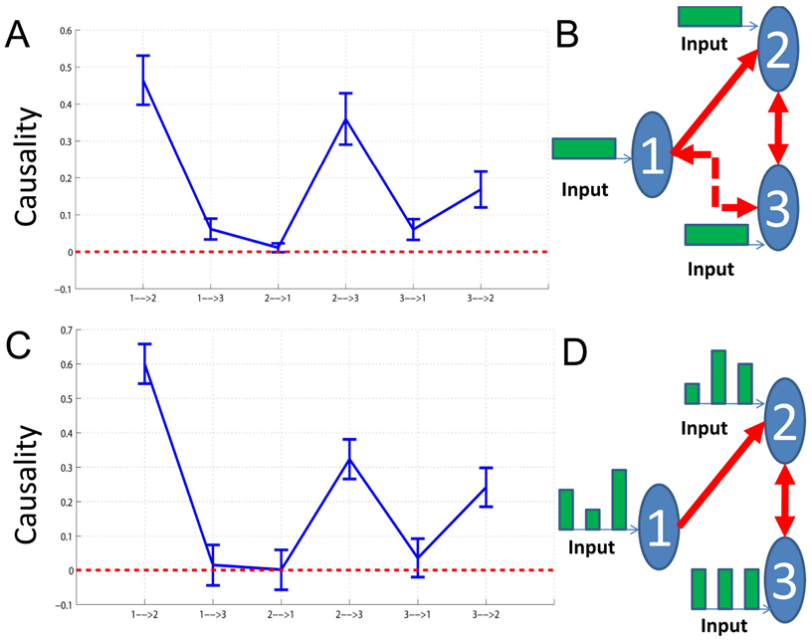
Results on Toy Model 2. Network structures with and without stimulus. A. Confidence intervals of all links between units. The data is generated with Eq. (15), but we use 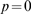 (without input) in our algorithms and a traditional VAR(10) model to detect the causal influence. B. The network structure of the state variables corresponding to A. Two additional causal relationships are marked by the dashed line. C. Confidence intervals of all links between units. The data is generated with Eq. (16) where 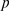 and 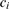, 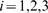 are generated with normal distribution (with input). D. The network structure of the state variables corresponding to C.

Here, we assumed that 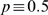 and the observation variables 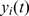, 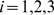 were identical to the state variables with observation noise. The variance of the noise was 0.1. It is obvious that the network structure is the same one as shown in Fig. 1B. However, if we ignore the constant input and just use a VAR model to detect this structure, we obtain the structure shown in Fig. 2A and Fig. 2B with confidence intervals where two additional causal relationships (i.e. 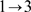 and 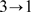) are presented showing that the real causal influence can no longer be correctly detected. Furthermore, when the input is not taken into consideration, the coefficients of the connection matrix will be meaningless and no longer provide us with the correct estimation of the strength of connection strengths between the state variables. This illustrates why we consider it necessary to incorporate the stimulus into our model although sometimes we don't know its form or intensity.

With our extended model we can, to some extent, solve the above issue and detect the causal influence correctly amongst those state variables affected by some unknown stimulus intermittently, although our model is originally set up for deterministic inputs.

We next generated a time series of 10000 time points which was composed of 10 segments with equal length, a.e., 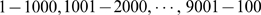. Each segment took the form:
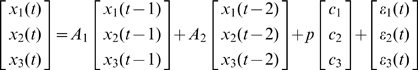
(16)where 
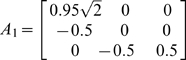
, 
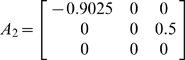
 and 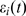, 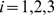 were zero mean uncorrelated Gaussian noise with variance 0.5, 0.8 and 0.6 respectively.

The five segments 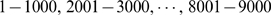 were generated according to the above toy model without input, i.e., 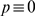, while the remaining five segments were assumed to include input of random intensity which would also affect the state variables randomly. Specifically, within each segment, 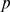 was assigned a random value which was generated with the normal distribution 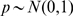, and the same was the case with 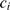: 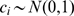, 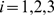. Observation variables were still assumed to be identical to the state variables with the variation of observation as 0.1.

Hence, the network structure of the three state variables is still the same as shown in Fig. 1B while each state variable is affected by some input that we don't know the intensity of. Fig. 2C and Fig. 2D show the predicted network structure with confidence intervals using our extended model. The results show that we can still detect the causal influence correctly in this situation.

### LFP from Left and Right Hemisphere

Local field potential data were obtained from 64-channel multielectrode arrays implanted in the right and left inferior temporal cortices of three sheep (one sheep only had electrodes in the right hemisphere) as previously described [13]. Recordings were made while the animals were presented with pairs of faces which they were required to discriminate between using an operant response in order to obtain a food reward. In between face pair presentations the animals were presented with a visual fixation stimulus (a white spot on a black screen). Recordings were made during sessions of 20–40 trials where they were either still learning the discrimination or had successfully achieved the learning criterion (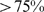 correct choice of rewarded face). In both the left and right IT the main oscillatory frequencies recorded are in the theta (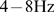) and gamma (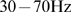) ranges and these two frequencies are coupled (theta phase and gamma amplitude) [13]. We have previously shown that learning increases theta amplitude, the ratio of theta to gamma, theta/gamma coherence and the tightness of theta phase [13].

**Figure 3:**
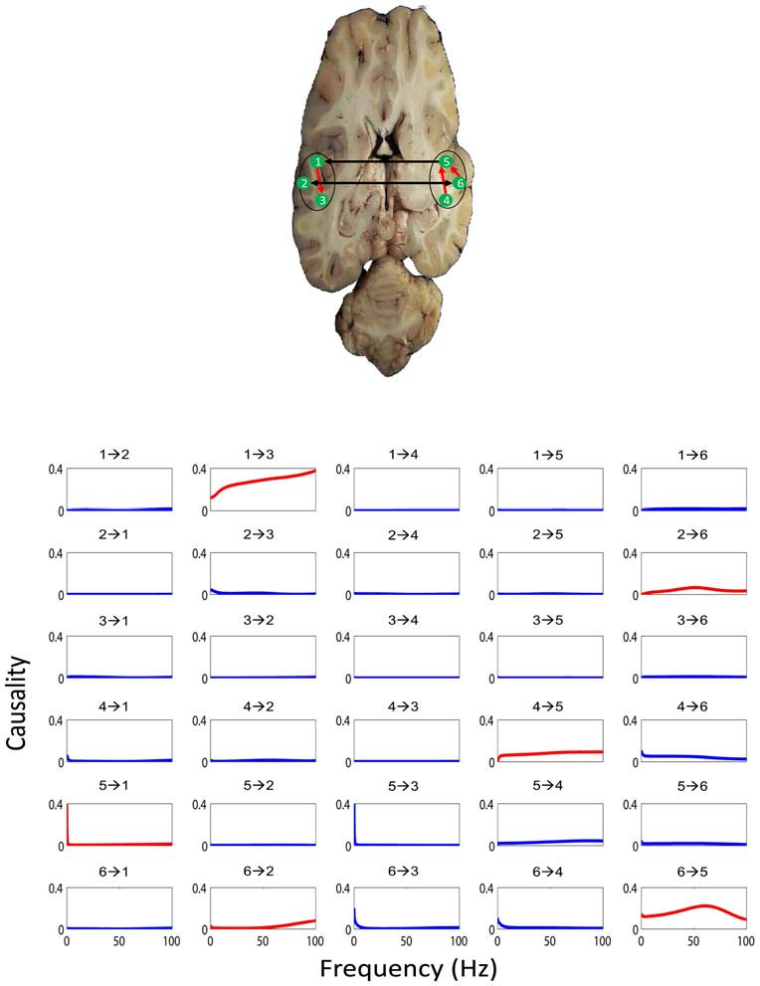
An example of the application of EGCM. The network detected by EGCM (top-panel) and the corresponding frequency decomposition (bottom-panel) for six randomly selected electrodes. In the frequency decomposition, significant causal influences are marked by red.

With these experimental data, we can directly use our EGCM to detect the global network for all electrodes in both brain hemispheres. However, due to the size of the network, there are at least a few thousand free parameters to fit. To avoid this issue, we adopt another approach here. For each session we randomly select 3 time series from each region respectively and apply our model to detect the network structure for the six electrodes. This procedure is repeated for 100 times for each session (see Fig. 3 for such an example). The visual stimulus to the IT (including feedforward and feedback signals) is impossible to know in the experiment. However, as we have shown above, we are able to make the assumption that the effect of the stimulus can be regarded as a constant input to each electrode. The inclusion of the stimulus signal will certainly make the model more reasonable.

A further problem here is that if we intend to reconstruct the connections for each six electrodes (left and right) before and after the stimulus respectively, we could end up with two different structures for the time series (not shown). This is certainly not the case since the duration of the stimuli is quite short (1–3 seconds) and the connections will not change in such a short time. To recover a reasonable structure of the connection in these areas in the brain, we therefore assume here that the connections in each trial don't change and the time series before and after the stimulus are generated from a unified structure. With the application of our EGCM approach, we can include the intermittent stimulus and obtain a comparatively reliable structure. Fig. 3 (top-panel) shows such an example where three electrodes in the left and three in the right are randomly selected and that inter- and intra-hemisphere interactions are detected. Fig. 3 (bottom-panel) is the corresponding frequency decomposition of the top panel.

**Figure 4:**
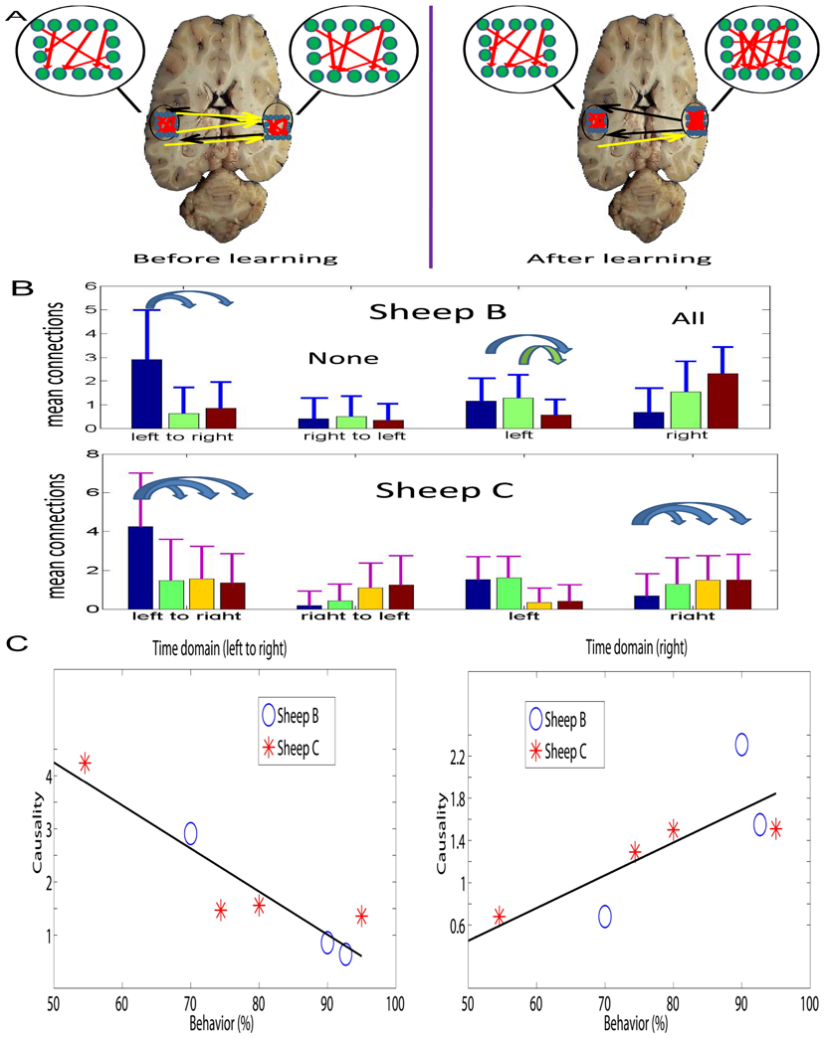
Asymmetry between left and right hemisphere in the time domain. A. A summary of the results in B, but locations in inferotemporal cortex are not precise, only for illustrative purposes. B. The mean connections from left hemisphere to right hemisphere, right hemisphere to left hemisphere and within both regions with the three bars corresponding to the results before learning (blue bar), after learning (green bar), and one month after learning (purple bar) in Sheep B. Significant changes after t-test are marked by arrows (right to left, all pairs are not significant, as indicated by “none”; within the right hemisphere, all pairs are significant, marked by “all”) . For Sheep C, an additional bar (one week after learning) is added (the third bar). Only significant changes from left to right and within the right hemisphere are indicated by arrows. C. Statistic summaries of results in B.

In Fig. 4 we show the mean connections within and between the left and right IT calculated using EGCM and as a function of learning. The results clearly demonstrate an asymmetry between the hemispheres. The top-panel is an illustration of the bottom panel which summarizes the results of all experimental data for the two sheep. The most noticeable change is a decrease in the number of connections from the left to the right and an increase in connections within the right but not in the left IT. Indeed there was a strong negative correlation between the number of left to right connections and the number within the right IT for both animals (Sheep B, 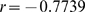 (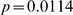); Sheep C, 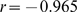 (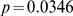)). These changes occurred as soon as the learning criterion was successfully achieved in successive blocks of trials (i.e. in as little as 5–10 minutes in the case sheep B where learning was successfully achieved within a specific recording session) and were maintained after 1 month or more post-learning. They were not simply the results of stimulus repetition because in Sheep B where recordings were made in repeated sessions of up to 120 trials but where the learning criterion was not achieved for one of the face pairs there were no connectivity changes observed.

**Figure 5:**
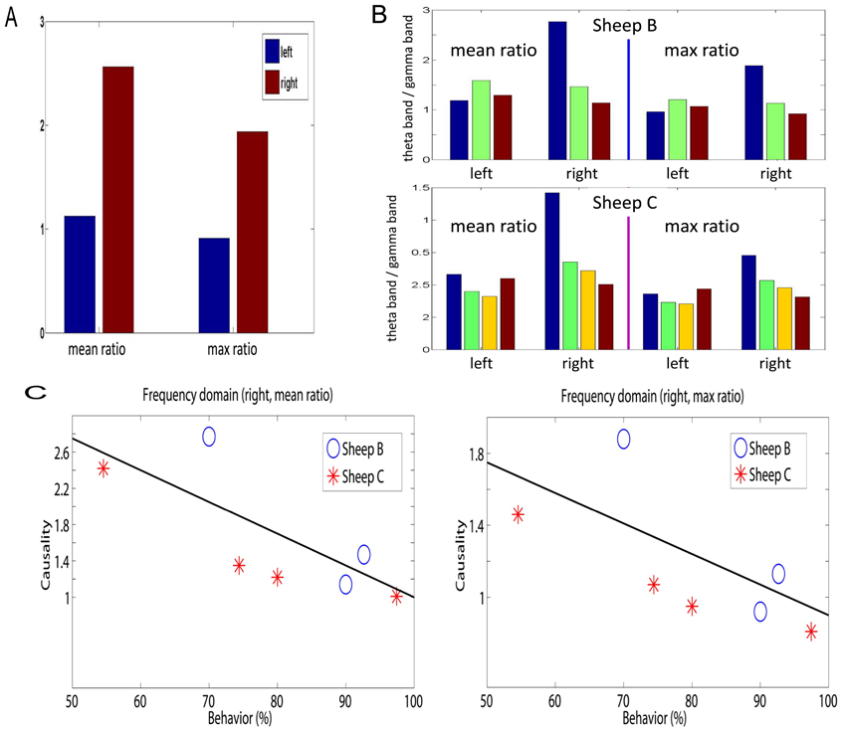
Asymmetry in the frequency domain interactions. A. Mean and maximum ratio using all the three sheep before and after learning. B. Upper panel: Mean and maximum ratio of sheep B (see Experiment subsection in Methods section) before learning, after learning and one month after learning (see Fig. 4). Bottom panel: Mean and maximum ratio of sheep C before learning (the first bar), immediately after learning (the second bar), one week after learning and one month after learning (the third and the fourth bar). C. Summaries of results in B.

One of the advantages of the extended approach is that we have a frequency domain decomposition. Brain rhythms, not surprisingly, have also been intensively investigated in the literature [35]. Here we concentrate on the two main frequency bands: theta band (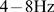) and gamma band (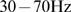) present in our IT recording data and which have been extensively linked to mechanisms of learning and memory [13],[35]. Using the frequency decomposition of our extended model discussed in Methods section, we looked at the following two quantities:
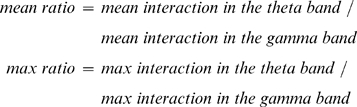

Fig. 5A shows the mean and maximum ratio integrating the data from all the three sheep in the experiment and at different stages of learning. From this it can be seen that both the mean and maximum ratios in the right hemisphere IT are about double those in the left hemisphere. This clearly indicates that for the right hemisphere, the theta band interaction is more dominant, i.e., the right hemisphere deals more with signals of lower frequency.

In order to provide a deeper insight into this frequency story, we compute the ratios at different stages of learning. Fig. 5B shows the mean and maximum ratio in two sheep before learning, after learning and a month after learning. An additional set of data is one week after learning for sheep C only. The most noticeable change is the reduction in the interactions in the theta band (low frequencies) in the right IT which occurs after learning and is maintained subsequently. Combining Fig. 4C (right) with Fig. 5C, we see that learning in general changes the connections in the right hemisphere (increasing), however, the increasing interactions are mainly due to the enhancement of the interaction at the high frequency domain.

## Discussion

### 

#### Comparing EGCM, GCM and DCM

EGCM has a strong connection with DCM as well as GCM. We consider the Dynamical Causal model:
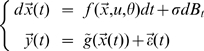
(17)where 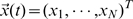 are state variables and 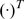 is the transpose of a vector, 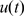 is a known deterministic input corresponding to designed experimental effects, 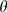 is the set of parameters to estimate, 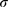 is the diffusion matrix (could depend on time) and 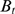 is the Brownian motion (or in general, it could be a martingale). The state variables 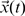 enter a specific model to produce the outputs 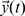 with the observation noise 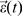.

Here we focus on the bilinear approximation of the Dynamical Causal model which is the most parsimonious but useful form [10]:
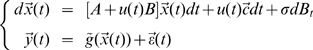
(18)where
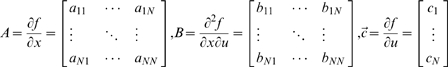
are parameters that mediate the intrinsic coupling among states, allow the inputs to modulate the coupling, and elicit the influence of extrinsic inputs on the states respectively. Here, for simplicity, we have expanded the state equation around 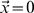 and assumed that 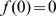.

The bilinear approximation of DCM is represented in terms of nonlinear differential equations while the GCM (see Eq. (1)) is formulated in discrete time and the dependencies among state variables are approximated by a linear mapping over time-lags which seems to be quite different. However, we can find the difference is that the bilinear form includes deterministic inputs and observation variables and equations which are not considered in GCM. The formulation (18) comes from the Volterra series and is certainly a more accurate and biophysical constraint representation of a biological system. On the other hand, the GCM with autoregressive representation always takes the past information into consideration while the bilinear approximation of DCM has no time-lags included in the differential equations although the general form of DCM may have [36]. So, if we alter the DCM to the form:
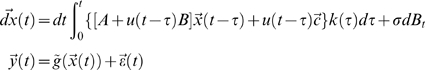
(19)where 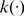 is a kernel function, then the DCM shares the feature of the GCM. On the other hand, our EGCM includes both deterministic inputs and observation variables thus takes the advantages of both DCM and GCM. In the general form of EGCM (Eq. (2)), we can find that when 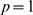, this is the discrete form of the bilinear form of DCM, and when 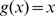, it is the GCM with additional inputs.

#### Advantage of extended approach

In contrast to all previous methods in estimating Granger causality in the literature where essentially a regression method is employed, in EGCM we incorporate noise observation variables and apply the extended Kalman filter to recover the state variables. Additional inputs are also included in EGCM on the basis of an autoregressive model. The advantage of such an approach over the previous methods is obvious. The EGCM is more reasonable when we are faced with experimental data affected by a particular stimulus and applicable to cases where we cannot track the state variables respectively but just a function of them, or where the observation noise is considerable. Comparing to the traditional VAR models, all the coefficients in EGCM correspond to intrinsic or latent dynamic coupling and changes induced by each input which endow the model with interpretability power. Furthermore, all the previous methods in estimating Granger causality are batch learning: they require collection of all data before an estimation can be made. The extended Kalman filter, on the other hand, is an online learning: we can now update Granger causality instantaneously. One may argue that this is a common feature of online learning vs. batch learning. However, it is novel in the context of Granger causality. When we are faced with biological data, this feature becomes particularly significant. As we know, adaptation, or learning in animals, is very important but this makes it difficult to analyze since adaptation introduces dynamic change into the system. The classical way of estimating Granger causality can cope with this difficulty by introducing sliding windows in analyzing data. Of course, to select an optimal window size is always an issue in such an approach. However, in Kalman filtering, we can have the advantage of the connection of the state variables from the connection matrix and such an issue is automatically resolved.

In comparison with the bilinear approximation of DCM, the advantages of EGCM are the following: First, it allows time delay in the model more naturally and easier to deal with. Time delay is ubiquitous in a biological system, no matter whether we are considering gene, protein, metabolic and neuronal networks. Secondly, using Granger causality we are able to summarize the causal effect into a single number which is more transparent and easy to understand, particularly in a system with a time delay. Thirdly, it allows a frequency domain decomposition. We know that when we are dealing with a dynamic system it is sometimes much informative to view it in the frequency domain rather than in the time domain, as we have partly demonstrated here. Of course, since Eq. (19) is a continuous time version of Eq. (2), the results in the frequency domain obtained for Eq. (2) is essentially for the DCM model as well. We summarize our comparisons in Table 1 (see [10]).

**Table 1:** Comparing DCM, GCM and EGCM.

Commonalities	DCM	GCM	EGCM
Multivariate analysis of time-series data	Yes	Yes	Yes
Models directed coupling	Yes	Yes	Yes
Inference on models	Yes	Yes	Yes
Frequency decomposition	Yes	Yes	Yes

Differences	DCM	GCM	EGCM
Causality based on temporal precedence	No	Yes	EGCM is more general
Causality based on control theory	Yes	No	Yes
Requires known inputs	Yes	No	In general yes, but see example 2
Requires orthogonal innovations	No	Yes	Not necessary
Requires stationary processes	No	Yes	Could use sliding window
Requires a specific biophysical model	Yes	No	Yes
Models non-linear coupling	Yes	No	Yes
Inference on model parameters	Yes	No	Yes

#### Other types of data

In the current paper, we have only applied EGCM to LFP data although it is clearly applicable to many other types of biological data. For example, in gene microarray data, we can have a readout of transcriptional changes in several thousand genes at different times over a period of many hours [37]. The same is the case with multiple protein measurements over time in biological systems or in metabolic changes. In all these situations estimation of altered causal connections in both time and frequency domains will provide invaluable information about changes occurring in the relationships between different components in the systems being studied.

#### IT hemispheric differences and learning

The results of the EGCM analysis of our IT LFP data provide the first evidence for connectivity changes between and within left and right ITs as a result of face recognition learning. It is clear that learning is a dynamic and complex process [38],[39]. In both sheep during learning there were more causal connections from the left to the right IT than vice-versa during learning trials. However, immediately after learning had occurred the number of left to right connections diminished to the same low level as seen from right to left. Within the hemispheres connectivity increased progressively over time in the right IT after learning but remained the same or decreased in the left IT. There was a strong negative correlation between the number of connections from left to right and the number within the right IT. This suggests that the left to right IT connections may exert some form of inhibitory control over the number within the right IT and that this therefore needs to be weakened for new face discriminations to be learned. The EGCM frequency analysis using theta and gamma oscillation data in the two hemispheres showed that during learning of new face pairs there was significantly more information being processed in the low frequency (theta) in the right IT than in the left. After learning however this declined and it appeared that the higher frequency information (gamma) became more dominant in both hemispheres. Lower frequency oscillations are more associated with global encoding over widespread areas of brain whereas higher frequencies are more associated with more localized encoding. This may suggest that during the course of learning new faces the right IT uses a more global mode of encoding to promote more rapid learning and that once learning has successfully occurred the right IT shifts to a more localized encoding strategy for maintaining learning. In the left IT on the other hand this more local encoding strategy predominates both during and after learning. This is in broad agreement with recent proposals that the left hemisphere is more involved in local encoding and the right in global encoding [18],[19] although in the case of face recognition it would appear that the right hemisphere shifts from a global to local encoding strategy once faces have been learned. Clearly more analyses of this kind are required before these differences in left and right brain hemispheres processing and interactions can be fully understood but combining multiple LFP recordings and EGCM will be a powerful future approach.

#### Field-type model

In the current paper, we have not explicitly introduced the spatio-correlation between each variables (electrodes). In other words, we have ignored the geometric relationship of electrodes in the array. This is certainly an over-simplification of the real situation due to the following reasons. First of all, despite the long history of multi-electrode array recordings, *in vivo* recording in, for example, IT is still very rare and difficult. Even we have a reliable recording session, the obtained data set is hard to fully analyze: for example, to reliably sort the spikes [40]. Secondly, assuming we could work out the spatio-temporal model for one animal, it is almost no sense to map the results about the detailed geometrical relationship (electrodes) to another animal. Also, in our experiments, we often face the situation that we have to discard the data from quite a few electrodes. A spatio-temporal (random field) approach as developed in fMRI [41] is not considered here. However, with the improvement of experimental techniques and the introduction of new techniques (see for example, functional multineuron calcium imaging [42]), a spatio-temporal model (a random field or a random point field) is cried for and is one of our future research topics. The well developed approach: dynamic expectation maximization [43], could play a vital role here.
